# Supplementary figures and images for: Evaluation of automated specialty palliative care in the intensive care unit: A retrospective cohort study
Source: PLoS One. 2021 Aug 11;16(8):e0255989. doi: 10.1371/journal.pone.0255989 (PMC8357176; doi:10.1371/journal.pone.0255989)

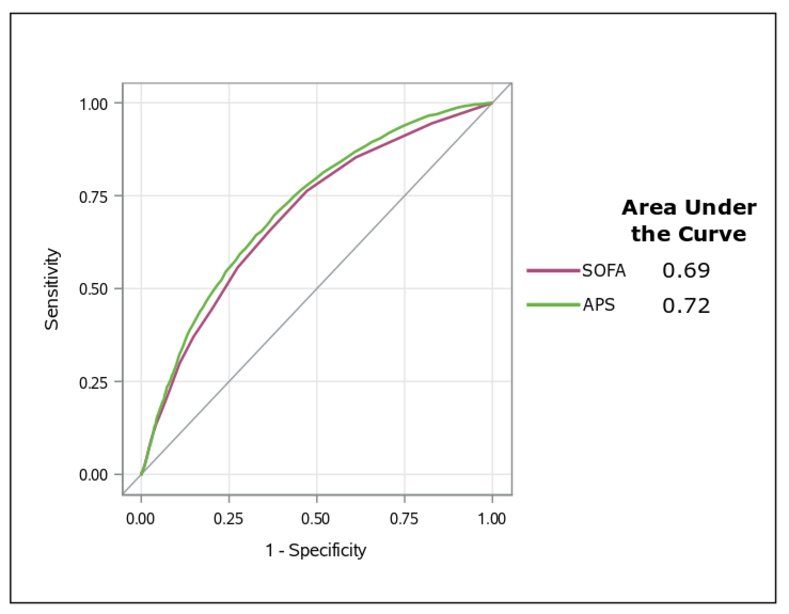

Supplement: S1 Fig — Abbreviations: APS = Acute Physiology Score, SOFA = Sequential Organ Failure Assessment. (TIF) [file pone.0255989.s001.tif]
