# Supplementary material for: Evaluation of automated specialty palliative care in the intensive care unit: A retrospective cohort study
Source: PLoS One. 2021 Aug 11;16(8):e0255989. doi: 10.1371/journal.pone.0255989 (PMC8357176; doi:10.1371/journal.pone.0255989)
Supplement: S1 Table — a T-test. b χ2 test of independence. c Statistics were calculated using available data. d Other Race includes American Indian or Alaska Native, Hispanic or Latinx, Native Hawaiian or other Pacific Islander, or Other. e Unknown Race includes Declined, Unable to answer, Unknown, or missing data. Abbreviations: APS = Acute Physiology Score, CCU = Cardiac Care Unit, CTICU = Cardiothoracic/Transplant Intensive Care Unit, ICU = Intensive Care Unit, MICU = Medical Intensive Care Unit, NSICU = Neurosciences-spine Intensive Care Unit, SICU = Surgical Intensive Care Unit, SOFA = Sequential Organ Failure Assessment. (DOCX) [file pone.0255989.s002.docx]

**S1 Table. Demographics, clinical characteristics, and hospitalization outcomes of all intensive care unit admissions, stratified by receipt of specialty palliative care consultation**

|  | All ICU admissions  N=48744 | All admissions that did not receive specialty palliative care  N=46779 | All admissions that received specialty palliative care  N=1965 | Mean difference or df, N | *P* value |
| --- | --- | --- | --- | --- | --- |
| Age in years, mean (95% CI) | 59.60 (59.44 – 59.75) | 59.38 (59.23 – 59.54) | 64.64 (63.92 – 65.36) | -5.25 (-5.99 – -4.52) | <.001^a^ |
| Female,^c^  No./total No. (%) | 21766/48744 (44.66) | 20848/46779 (44.56) | 918/1965 (46.72) | df=1, N=48736 | 0.061^b^ |
| Race,  No./total No. (%) |  |  |  | df=4, N=48744 | <.001^b^ |
| White or Caucasian | 28359/48744 (58.18) | 27328/46779 (58.42) | 1031/1965 (52.47) |  | - |
| Black or African American | 10215/48744 (20.96) | 9816/46779 (20.98) | 399/1965 (20.31) |  | - |
| Asian | 1437/48744 (2.95) | 1370/46779 (2.93) | 67/1965 (3.41) |  | - |
| Other Race^d^ | 4513/48744 (9.26) | 4298/46779 (9.19) | 215/1965 (10.94) |  | - |
| Unknown Race^e^ | 4220/48744 (8.66) | 3967/46779 (8.48) | 253/1965 (12.88) |  |  |
| Hispanic or Latinx Ethnicity,  No./total No. (%) | 4216/48744 (8.65) | 4071/46779 (8.70) | 145/1965 (7.38) | df=2, N=48744 | <.001^b^ |
| Illness severity, mean (95% CI) |  |  |  |  |  |
| APS Score | 37.96 (37.75 – 38.16) | 37.22 (37.01 – 37.42) | 55.27 (54.07 – 56.46) | -18.05 (-19.26 – -16.84) | <.001^a^ |
| SOFA score | 3.35 (3.33 – 3.38) | 3.26 (3.23 – 3.29) | 5.69 (5.51 – 5.86) | -2.43 (-2.61 – - 2.26) | <.001^a^ |
| ICU type,  No./total No. (%) |  |  |  | df=4, N=48744 | <.001^b^ |
| MICU | 14942/48744 (30.65) | 13924/46779 (29.77) | 1018/1965 (51.81) |  | - |
| NSICU | 11266/48744 (23.11) | 10947/46779 (23.40) | 319/1965 (16.23) |  | - |
| CTICU | 9172/48744 (18.82) | 8967/46779 (19.17) | 205/1965 (10.43) |  | - |
| SICU | 7237/48744 (14.85) | 7106/46779 (15.19) | 131/1965 (6.67) |  | - |
| CCU | 6127/48744 (12.57) | 5835/46779 (12.47) | 292/1965 (14.86) |  | - |
| ICU length of stay in days, mean (95% CI) | 3.50 (3.44 – 3.56) | 3.23 (3.18 – 3.28) | 9.93 (9.29 – 10.57) | -6.70 (-7.35 – -6.05) | <.001^a^ |
| In-hospital death,  No./total No. (%) | 4131/48744 (8.47) | 3082/46779 (6.59) | 1049/1965 (53.38) | df=1, N=48744 | <.001^b^ |

^a^ T-test

^b^ χ^2^ test of independence

^c^ Statistics were calculated using available data.

^d^ Other Race includes American Indian or Alaska Native, Hispanic or Latinx, Native Hawaiian or other Pacific Islander, or Other

^e^ Unknown Race includes Declined, Unable to answer, Unknown, or missing data

Abbreviations: APS= Acute Physiology Score, CCU= Cardiac Care Unit, CTICU= Cardiothoracic/Transplant Intensive Care Unit, ICU= Intensive Care Unit, MICU= Medical Intensive Care Unit, NSICU= Neurosciences-spine Intensive Care Unit, SICU= Surgical Intensive Care Unit, SOFA= Sequential Organ Failure Assessment
