# Supplementary material for: Evaluation of automated specialty palliative care in the intensive care unit: A retrospective cohort study
Source: PLoS One. 2021 Aug 11;16(8):e0255989. doi: 10.1371/journal.pone.0255989 (PMC8357176; doi:10.1371/journal.pone.0255989)
Supplement: Attachment — Submitted filename: Response to Reviewers.docx [file pone.0255989.s003.docx]

**Editor comments**

1. Please ensure that your manuscript meets PLOS ONE's style requirements, including those for file naming.

*Author response: We have reviewed our manuscript and made updates as appropriate to ensure the manuscript meets PLOS ONE’s style requirements.*

2. Please review your reference list to ensure that it is complete and correct. If you have cited papers that have been retracted, please include the rationale for doing so in the manuscript text or remove these references and replace them with relevant current references. Any changes to the reference list should be mentioned in the rebuttal letter that accompanies your revised manuscript. If you need to cite a retracted article, indicate the article’s retracted status in the References list and also include a citation and full reference for the retraction notice.

*Author response: We have reviewed the reference list for completeness and accuracy. We have not made any changes to the reference list.*

3. We noted in your submission details that a portion of your manuscript may have been presented or published elsewhere.

[A portion of this work was published as an American Thoracic Society International Conference abstract.]

Please clarify whether this conference proceeding was peer-reviewed and formally published. If this work was previously peer-reviewed and published, in the cover letter please provide the reason that this work does not constitute dual publication and should be included in the current manuscript.

*Author response**: An abstract representing a portion of these analyses was peer-reviewed and accepted for presentation by the American Thoracic Society International Conference in 2020. The abstract was published in the American Thoracic Society 2020 International Conference Abstract Issue of the American Journal of Respiratory and Critical Care Medicine. The abstract contained only a small portion of work described in this manuscript. The manuscript expands upon the results presented in abstract and contains data that are not presented in the abstract. We have updated the cover letter to reflect this explanation.*

4. Please include captions for your Supporting Information files at the end of your manuscript, and update any in-text citations to match accordingly.

*Author response: We have updated the manuscript to include captions for the supporting information and updated any in-text citations to match accordingly.*

**Reviewer comments**

Reviewer's Responses to Questions

1. Is the manuscript technically sound, and do the data support the conclusions?

The manuscript must describe a technically sound piece of scientific research with data that supports the conclusions. Experiments must have been conducted rigorously, with appropriate controls, replication, and sample sizes. The conclusions must be drawn appropriately based on the data presented.

Reviewer #1: Yes

Reviewer #2: Yes

Reviewer #3: Yes

2. Has the statistical analysis been performed appropriately and rigorously?

Reviewer #1: Yes

Reviewer #2: Yes

Reviewer #3: Yes

3. Have the authors made all data underlying the findings in their manuscript fully available?

The PLOS Data policy requires authors to make all data underlying the findings described in their manuscript fully available without restriction, with rare exception (please refer to the Data Availability Statement in the manuscript PDF file). The data should be provided as part of the manuscript or its supporting information or deposited to a public repository. For example, in addition to summary statistics, the data points behind means, medians and variance measures should be available. If there are restrictions on publicly sharing data—e.g. participant privacy or use of data from a third party—those must be specified.

Reviewer #1: Yes

Reviewer #2: Yes

Reviewer #3: No

*Author response: The raw data from this study cannot be shared publicly because they contain potentially identifying and sensitive patient information derived from electronic health records. Data requests by researchers who meet criteria for access to confidential data can be directed to the Northwestern University Institutional Review Board (contact via email at irb@northwestern.edu).*

4. Is the manuscript presented in an intelligible fashion and written in standard English?

PLOS ONE does not copyedit accepted manuscripts, so the language in submitted articles must be clear, correct, and unambiguous. Any typographical or grammatical errors should be corrected at revision, so please note any specific errors here.

Reviewer #1: Yes

Reviewer #2: Yes

Reviewer #3: Yes

Reviewer Comments to the Author

Reviewer #1: very relevant data. Your study needs to include later other centers furthermore it is I think very interesting to study different categories of patients like patients COVID 19 infected as this subject is a big field of research and evaluation

*Author response: Thank you for your comments. We agree that future research should examine the use of specialty palliative care triggers in different patient populations such as those with COVID-19. We also agree that future work should explore the use of specialty palliative care triggers across multiple medical centers.*

Reviewer #2: The study is well-conducted and the conclusions are appropriate. The authors show the need for new triggers for automated specialty palliative care in ICU. Further investigation could be useful in assessing the effectiveness of the implementation with these new triggers.

*Author response: Thank you for your comments. We agree that future work should involve development and implementation of more patient-centered triggers to better identify patients who may benefit from specialty palliative care.*

Reviewer #3:

1. This manuscript by Secunda and coworkers reports the results of a retrospective cohort study in the intensive care unit (ICU) setting. The study aimed to examine whether the most commonly used Specialty Palliative Care (SPC) triggers accurately represent actual SPC consult in the ICU at an academic center. Using SPC triggers alone may miss subsets of patients who may otherwise benefit from SPC (e.g., younger patients). The authors state that the mortality-based SPC triggers used in this study are ones most commonly used and discussed in literature, which include: ICU admission following hospital stay >= 10 days, age >80 with 2 or more life-threatening comorbidities, stage 4 malignancy, post cardiac arrest, and intracerebral hemorrhage requiring mechanical ventilation.

The authors found that these conventional mortality-based criteria for SPC triggers have poor sensitivity and specificity for actual SPC consults. They did acknowledge that they cannot evaluate the appropriateness of the actual SPC utilization due to inability to measure whether the patient’s palliative care need was truly met.

The implications and future directions of this study were well stated in the discussion: to develop a more patient-centered predictive model to better and earlier identify patients who may benefit from SPC.

2. Minor comments

 a.) Please follow the STROBE guideline for reporting more closely. Use the predefined headers.

*Author response: We appreciate your comment and have updated the manuscript to follow the STROBE guidelines more closely.*

b.) The IRB approval statement typically appears in the first paragraph of the methods.

*Author response: We appreciate your comment and have moved the IRB approval statement to the first paragraph in the methods section in the revised manuscript.*

c.) Line 101-102: “Multiple reasons for consultation could be used for single SPC encounter, and some notes did not document an explicit reason for consultation.” How were these data treated?

*Author response: We appreciate the opportunity to further clarify how we categorized reasons for specialty palliative care consultation. We have updated the methods section in the revised manuscript with the following clarification statement:*

If an SPC note included more than one reason for consultation, all reasons were abstracted and included in the analysis. If an SPC did not contain any stated reason for consultation, the encounter was designated as “no evident reason for consultation”, which was categorized as “other” during analyses.

d.) Table 1: Why is the denominator for race in all 3 columns not corresponding to the total number of subjects? For example, the first column under race shouldn’t the denominator be 1965 instead of 1896? What happened to the missing numbers? Could be due to “unknown” category? If so, would add an “unknown” category for race.

*Author response: Thank you for pointing out this additional opportunity to clarify our results. In the dataset used in this study, “Unknown” is a selection made in the electronic medical record for the demographic variables race and ethnicity. “Missing” signifies that data is absent for these variables. We originally treated “unknown” differently than missing data which is why the original denominator was 1896 (which excluded the admissions missing race and/or ethnicity data). However, upon further consideration of these categories based on your comments, we agree that missing race and ethnicity data should be treated the same as “unknown” in a separate category. Thus, we have updated table 1 and the supplementary table to reflect the following new categories for race: (1) White or Caucasian, (2) Black or African American, (3) Asian, (4) Other Race (which includes American Indian or Alaska Native, Hispanic or Latinx Race, Native Hawaiian or other Pacific Islander, and Other), and (5) Unknown Race (which includes declined, unable to answer, unknown, or missing data). We have also updated the statistics to reflect this change which includes the unknown category (which is a combination of the available data stating that race is unknown and missing data). Please see the revised manuscript, Table 1, and Supplemental Table for specific tracked changes.*
